# Distribution of causes of maternal mortality among different socio-demographic groups in Ghana; *a descriptive study*

**DOI:** 10.1186/1471-2458-11-159

**Published:** 2011-03-10

**Authors:** Benedict O Asamoah, Kontie M Moussa, Martin Stafström, Geofrey Musinguzi

**Affiliations:** 1 International Master Programme in Public Health, Faculty of Medicine, Lund University, Malmö University Hospital, Malmö, Sweden; 2 Department of Clinical Sciences Malmö, Division of Social Medicine and Global Health, Lund University, Malmö University Hospital, Malmö, Sweden

## Abstract

**Background:**

Ghana's maternal mortality ratio remains high despite efforts made to meet Millennium Development Goal 5. A number of studies have been conducted on maternal mortality in Ghana; however, little is known about how the causes of maternal mortality are distributed in different socio-demographic subgroups. Therefore the aim of this study was to assess and analyse the causes of maternal mortality according to socio-demographic factors in Ghana.

**Methods:**

The causes of maternal deaths were assessed with respect to age, educational level, rural/urban residence status and marital status. Data from a five year retrospective survey was used. The data was obtained from Ghana Maternal Health Survey 2007 acquired from the database of Ghana Statistical Service. A total of 605 maternal deaths within the age group 12-49 years were analysed using frequency tables, cross-tabulations and logistic regression.

**Results:**

Haemorrhage was the highest cause of maternal mortality (22.8%). Married women had a significantly higher risk of dying from haemorrhage, compared with single women (adjusted OR = 2.7, 95%CI = 1.2-5.7). On the contrary, married women showed a significantly reduced risk of dying from abortion compared to single women (adjusted OR = 0.2, 95%CI = 0.1-0.4). Women aged 35-39years had a significantly higher risk of dying from haemorrhage (aOR 2.6, 95%CI = 1.4-4.9), whereas they were at a lower risk of dying from abortion (aOR 0.3, 95% CI = 0.1-0.7) compared to their younger counterparts. The risk of maternal death from infectious diseases decreased with increasing maternal age, whereas the risk of dying from miscellaneous causes increased with increasing age.

**Conclusions:**

The study shows evidence of variations in the causes of maternal mortality among different socio-demographic subgroups in Ghana that should not be overlooked. It is therefore recommended that interventions aimed at combating the high maternal mortality in Ghana should be both cause-specific as well as target-specific.

## Background

Maternal mortality remains as a major Public Health challenge despite numerous strategies devised by the international community to curb it. Globally, maternal mortality is the leading cause of death among females aged 15-49 years old. More than 1500 women die each day from pregnancy related causes resulting in an estimated 550 000 maternal deaths annually [1]. In 2010, estimates developed by the WHO, UNICEF, UNFPA and the World Bank [2] suggest that worldwide, about 260 women die per 100 000 live births and most of these deaths occur in sub-Saharan Africa. These estimates indicate that Africa recorded the highest Maternal Mortality Ratio (MMR) of 620 per 100 000 live births, whilst Europe recorded the lowest MMR of 21 maternal deaths per 100 000 live births. Globally, Greece recorded the lowest maternal deaths by country with 2 per 100 000 live births compared with the alarmingly high MMR of 1400 deaths per 100 000 live births in Afghanistan [2]. In sub-Saharan Africa, Cape Verde recorded the lowest MMR of 94 whilst Chad and Somalia recorded the highest MMR of 1200 [2]. These figures show a very large discrepancy in maternal health, with sub-Saharan Africa experiencing the poorest outcome.

To respond to this challenge, the Millennium Development Goal 5 (MDG 5), which aims to improve maternal health was developed. The target is to reduce by three-quarters the MMR between 1990 and 2015 and achieve universal access to reproductive health care by 2015. A study by Hogan and colleagues, in 2010, found that there was a decrease in the global MMR estimates from 320 in 1990 to 251 in 2008 per 100 000 live births [3]. Even in the presence of this change, very few countries are on track to achieve MDG 5 [3]. This stagnation calls for different innovations and strategies to tackle this global menace.

A study by Thonneau *et al.*, (2004) [4] carried out in twelve maternities in Benin, Ivory Coast and Senegal, found that hypertensive disorders and post-partum haemorrhage caused 29% and 15% respectively of maternal mortalities in these three African countries. These were the highest causes of maternal mortality among this group [4]. Inconsistency in clinical diagnosis of the causes of maternal deaths has also been reported as a possible reason for why this challenge remains unabated [5]. Infectious diseases related to maternal mortalities are often under-diagnosed whilst hypertensive disorders related to pregnancy (including Eclampsia) are in most cases, over-diagnosed [6].

Ghana's MMR continues to be unacceptably high despite efforts made in an attempt to meet MDG 5. The Ministry of Health has been called on to treat maternal mortality as a national emergency [7]. Estimation of Maternal Mortality Ratio in Ghana varies widely by source and method of estimation [8]. Figures from the WHO, UNICEF and UNFPA for Ghana indicate 740 maternal deaths in 1990, 590 in 1995, 540 in 2000 and 560 in 2005 per 100 000 live births [9,10]. This contrasts lower estimation from the Ghana Statistical Service which were 214 in 1992 and 378 per 100 000 live births between 2000 and 2005 [11]. This high level of uncertainty and discrepancy makes MMR unsuitable for monitoring maternal mortality/maternal health trends in short term [12].

The causes of maternal mortality are usually sub-grouped into direct obstetric and indirect causes. Direct causes of maternal mortality as indicated in previous studies conducted in Ghana include haemorrhage (postpartum and ante partum), abortion, miscarriage, sepsis, obstructed labour, ectopic pregnancy, (Pre-) eclampsia and embolism [11,13-15]. The indirect causes of maternal mortality are mostly infectious and non-infectious diseases and other miscellaneous causes. These indirect causes include mainly malaria, HIV/AIDS, hepatitis, respiratory infections, anaemia, sickle cell disease, meningitis, cerebrovascular diseases and others [11,13-15].

In Ghana, several interventions targeting the reduction of maternal mortality have been implemented. Notable among these is the user fee exemption policy instituted in 2003. This policy exempts all pregnant women from paying for delivery costs at public, mission and private health facilities [16]. Evaluation of this intervention between 2003 and 2006 showed dramatic reduction of direct maternal deaths but no significant impact on indirect maternal deaths [17]. Thus, maternal mortality can be prevented in many cases but this demands not only a comprehensive understanding of the causes, but also, more importantly, an understanding of how the different causes are distributed in various groups with different characteristics. A number of studies have been conducted on maternal mortality in Ghana [8,11,13-15,18,19]; however, only one [11] attempted to analyze causes of maternal mortality with respect to socio-demographic groups, and did so with limited detail. The study reported percentage distribution of maternal deaths by cause of death, according to age and region. Much emphasis was put on abortion in the analysis. The study did not report the maternal mortality cause-specific risks associated with the different socio-demographic groups. Thus, detailed analysis of the causes of these mortalities, stratified by various socio-economic and demographic characteristics, is essential for formulating specific interventions to deal with these causes in different socio-demographic groups. This would help to accelerate Ghana and other similar nations in sub-Saharan Africa, towards the realization of Millennium Development Goal 5. The aim of the study is, therefore, to assess and analyse the causes of maternal mortality according to socio-demographic factors in Ghana.

## Method

### Data collection

**Figure 1:**
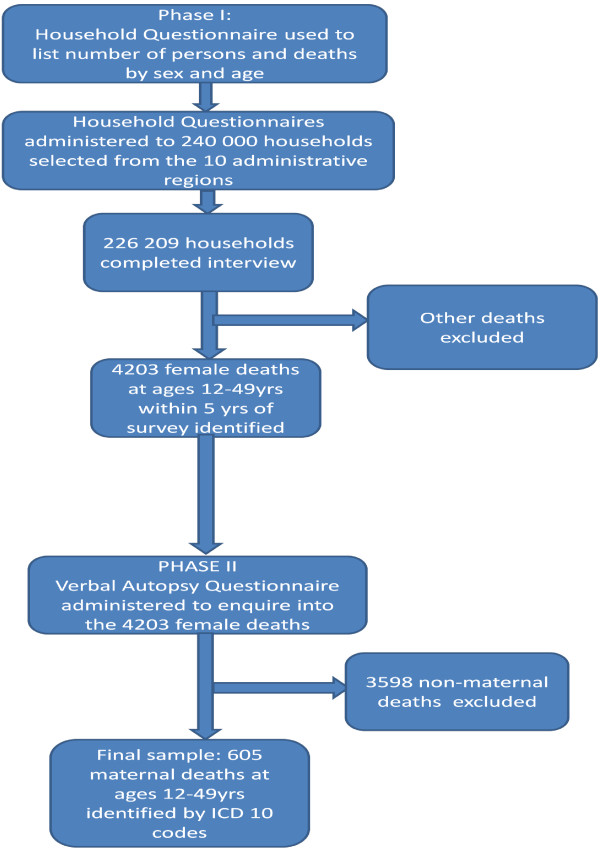
**Flow chart on data collection process**.

The data for this study was extracted from the Ghana Maternal Health Survey 2007, which was acquired from the Ghana Statistical Service. Figure 1 illustrates the data collection process. The primary data was gathered in a two-phase fieldwork. In phase I, a nationally representative sample of 240 000 households were selected from the 10 administrative regions of Ghana across urban and rural areas, out of which 226 209 completed the household questionnaires. The household questionnaire was used to list number of persons and deaths in a household by age and sex in the five years that preceded the survey. For female deaths, additional questions were introduced. This included: whether death occurred at ages 12-49; whether the woman was pregnant at death; whether she died during childbirth; and whether she died within two months of delivery. The purpose of the household questionnaire was to identify the target households for the administration of the Verbal Autopsy Questionnaire in phase II.

Households that reported one or more deaths of women aged 12-49 years in the five years that preceded the phase I survey were followed-up in phase II to complete a verbal autopsy questionnaire. 4203 female deaths (maternal and non-maternal) were identified during phase II. The final causes of the identified deaths were classified according to the International Statistical Classification of Diseases and Related Health Problems (ICD-10) [20]. Of the 4203 female deaths, 605 were maternal deaths in the age group 12-49 years, the sample that was used in this study.

Figure 1: Flow chart on data collection process.

### Definition of variables

*Outcome/Dependent variables*: *cause of maternal mortality*. This involves causes related to pregnancy, childbirth and the puerperium according to the ICD-10 codes [20]. The final causes of death according to ICD-10 codes were grouped into 9 categories. The five main variables used in the logistic regression analysis were:

*a) Haemorrhage*, which includes both ante partum and postpartum haemorrhage; *b)Abortion*, which represents all medical, attempted, failed, unspecified and other forms of abortion according to ICD-10 [20]; *c)Other infectious diseases*, which mainly comprised malaria and other protozoa diseases, viral hepatitis and tuberculosis; *d) Other non-infectious diseases*, contained pregnancy-related deaths from anaemia and diseases of the respiratory, circulatory and digestive systems; *e) Miscellaneous*: which contained maternal deaths from obstetric deaths of unspecified causes, rupture of uterus, complications of obstetric surgery, embolism, complications of anaesthesia, obstetric shock and other complications of labour and delivery.

Other variables that were described but not used in the logistic regression analysis were; hypertensive disorders of pregnancy (*including Eclampsia*), sepsis, obstructed labour and miscarriage (*this refers to all forms of spontaneous abortions*).

*Independent Variables*: The independent variables in this study were

1. *Maternal age at death*: Maternal age was categorised into eight groups of 12-14years, 15-19years, 20-24years, 25-29years, 30-34years, 35-39years, 40-44years and 44-45years.

2. *Educational level*: This was put into four categories

a. Never attended: those who confirmed to have never attended any formal educational system as well as those whose educational levels were unknown.

b. Basic education: those with some level of formal education up to 9 years. The category represented women with primary, middle school or junior secondary school education.

c. Senior high school: those women with up to 12 years of formal education or whose education ended at the senior secondary/high school level.

d. Tertiary or higher education: women who completed at least 15 years of formal education and included those who completed training college, polytechnic or university level.

3. *Residence*: Residence was coded in two categories that described the urban/rural residence status of the deceased.

4. *Marital status*: Marital status was put into two categories

a. Single: those who never married, or were separated, divorced, or widowed.

b. Married: those who were married and those living with partner at the time of death.

### Measurement of variables and statistical methods

The causes of death were put into two main categories labelled as

1. Maternal deaths (between 12-49 years): These were true maternal deaths defined as *the death of a woman during pregnancy or within 42 days of the end of pregnancy from causes related to or aggravated by pregnancy, but not from incidental causes*

2. Non-maternal deaths: Non-pregnancy related female deaths.

The data was then split according to these categories and maternal deaths were analysed. A frequency table showing the distribution of causes of maternal mortality according to age, educational level, residence and marital status was computed. Each of the causes under the nine categories of maternal mortality was dichotomised and cross-tabulated with the above socio-demographic variables to analyse how these causes differ in the different groups. Logistic Regression analysis was then carried out on the top five causes, using each dichotomised cause of mortality as the dependent variable. Age group, educational level, residence and marital status were used as the predictor variables/covariates. A crude odds ratio (OR) was computed with 95% Confidence Interval (CI) using one covariate at a time. An adjusted Odds Ratio (aOR) was also computed using one covariate at a time and adjusting for all the other variables in one model. Statistical software PASW-statistics (SPSS) 18.0 was used for the analysis.

## Results

The mean age of women who died from pregnancy-related causes was 29.3years (SD 7.6). Table 1 shows how maternal deaths are distributed according to age group, educational level, marital status and residence status. The results (Table 1) show an inverse trend in maternal mortalities with increasing educational level. Thus 54.9% of maternal deaths occurred in women whose education ended at the basic level whilst 2.1% were ascribed to those whose education ended at the tertiary/higher level. Most maternal deaths occurred among people that resided in rural areas (64.1%) compared to those in urban residence (35.9%) as well as the married (83.6%) compared to single women (16.4%).

**Table 1:** Socio-demographic characteristics of 605 women who died from pregnancy-related causes in Ghana between 2000 and 2005

Variable	Number	Valid percent (%)
**Age group(years)**		
12-14	3	0.5
15-19	62	10.2
20-24	115	19.0
25-29	133	22.0
30-34	116	19.2
35-39	106	17.5
40-44	55	9.1
45-49	15	2.5
**Total**	**605**	**100**

**Highest Educational level**		
Never Attended	208	34.4
Basic Education	332	54.9
Senior High School	52	8.6
Tertiary/Higher Education	13	2.1
**Total**	**605**	**100**

**Residence**		
Urban	216	35.9
Rural	389	64.1
**Total**	**605**	**100**

**Marital Status**		
Single	99	16.4
Married	506	83.6
**Total**	**605**	**100**

Table 2 shows the causes of maternal mortality. Haemorrhage (22.8%) was the highest cause of maternal mortality. The other top causes were infectious diseases (13.9%), abortion (13.7%), miscellaneous (13.6%) and other non-infectious diseases (12.4%). Maternal deaths were highest in the age group 25-29years (22.0%) followed by 30-34years (19.2%) and 20-24years (19.0%).

**Table 2:** Causes of maternal mortality among 605 women who died from pregnancy-related causes in Ghana between 2000 and 2005

Variable	Number	Valid percent (%)
**Direct causes**		
Haemorrhage(ante partum and postpartum)	138	22.8
Abortion(Medical, Attempted, failed, other, unspecified)	83	13.7
Hypertensive disorders of pregnancy(including Eclampsia)	54	8.9
Sepsis	42	6.9
Obstructed Labour	27	4.5
Miscarriage	20	3.3
**Indirect causes**		
Other Infectious diseases*	84	13.9
Other non-infectious diseases**	75	12.4
Miscellaneous***	82	13.6
**Total**	**605**	**100**

*The major infectious diseases were malaria 53.6%, viral hepatitis 13.1%, unspecified infections 7.1% and tuberculosis 2.4%.

** The major cause of death in the category of non-infectious diseases was Anaemia 41.3%; followed by diseases of blood and blood-forming organs 17.3%; respiratory diseases 14.6%; and circulatory diseases 12.0%.

*** Miscellaneous causes comprised mainly obstetric deaths of unspecified causes 26.8%, rupture of uterus 17.1%, complications of obstetric surgery 14.6%, embolism 9.8%, complications of anaesthesia 4.8% and other complications of pregnancy, labour and puerperium.

Table 3 shows how the causes of maternal mortality are distributed with respect to age group, educational level, marital status and regional difference. Haemorrhage is highest in the age group 35-39 years (27.5%), followed by 30-34 years (22.5%) 25-29 years (20.3%) and 20-24 years (14.5%). In contrast, abortion is highest in the age group of 20-24 years (26.5%), followed by 15-19 years (20.5%), 30-34 years (16.5%) and 25-29 years (13.3%). Additionally, 28.6% of deaths from infectious diseases related to pregnancy occurred in the age group 20-24 years; 22.6% in the age group 25-29 years; 17.9% in the age group 30-34 with only 6.0% occurring in the age range of 40-44 years. There was no death from infectious diseases related to pregnancy within the age group 44-49 years. The major infectious diseases that caused pregnancy-related deaths were malaria 53.6%, viral hepatitis 13.1%, unspecified infections 7.1% and tuberculosis 2.4%. Deaths from miscellaneous causes were common between the ages of 25 and 44years (see Table 3). Miscellaneous causes comprised mainly obstetric deaths of unspecified causes 26.8%, rupture of uterus 17.1%, complications of obstetric surgery 14.6%, embolism 9.8%, complications of anaesthesia 4.8% and other complications of pregnancy, labour and puerperium. Maternal deaths from non-infectious diseases were highest among 25-29years (32.0%) and 20-24years old women (21.3%). Anaemia 41.3% was the major cause of death in the category of non-infectious diseases followed by diseases of blood and blood-forming organs 17.3%; respiratory diseases 14.6%; and circulatory diseases 12.0%.

**Table 3:** Variations in the causes of maternal mortality according to Socio-demographic characteristics of 605 women who died from pregnancy-related deaths in Ghana between 2000 and 2005

Variables	Haemorrhage (*Ante partum and Postpartum*) n (%)	Other Infectious Diseases n (%)	Abortion (*Medical, Attempted, failed, other, unspecified*) n (%)	Miscellaneous n (%)	Other non-Infectious diseases n (%)
**Age group(years)**					
12-14	-	-	2(2.4)	-	-
15-19	7(5.1)	9(10.7)	17(20.5)	8(9.8)	8(10.7)
20-24	20(14.5)	24(28.6)	22(26.5)	10(12.2)	16(21.3)
25-29	28(20.3)	19(22.6)	11(13.3)	15(18.3)	24(32.0)
30-34	31(22.5)	15(17.9)	14(16.9)	17(20.7)	9(12.0)
35-39	38(27.5)	12(14.3)	5(6.0)	17(20.7)	11(14.7)
40-44	12(8.7)	5(6.0)	7(8.4)	14(17.1)	5(6.7)
45-49	2(1.4)	0(0.0)	5(6.0)	1(1.2)	2(2.7)
**Total**	138(100)	84(100)	83(100)	82(100)	75(100)

**Educational level**					
Never Attended	53(38.4)	37(44.0)	23(27.7)	28(34.1)	33(44.0)
Basic Education	72(52.2)	39(46.4)	49(59.0)	45(54.9)	34(45.3)
Senior High Sch.	10(7.2)	7(8.3)	10(12.0)	7(8.5)	6(8.0)
Tertiary/Higher	3(2.2)	1(1.2)	1(1.2)	2(2.4)	2(2.7)
**Total**	138(100)	84(100)	83(100)	82(100)	75(100)

**Residence**					
Urban	43(31.2)	29(34.5)	28(33.7)	31(37.8)	25(33.3)
Rural	95(68.8)	55(65.5)	55(66.3)	51(62.2)	50(66.7)
**Total**	138(100)	84(100)	83(100)	82(100)	75(100)

**Marital Status**					
Single	9(6.5)	9(10.7)	35(42.2)	14(17.1)	13(17.3)
Married	129(93.5)	75(89.3)	48(57.8)	68(82.9)	62(82.7)
**Total**	138(100)	84(100)	81(100)	82(100)	75(100)

- No data

In all the causes of maternal mortality as seen in Table 3, mortality decreased with increasing educational level. Mortality was generally high for women who lived in rural areas as opposed to urban residence. In all the causes of maternal mortality, percentage of married women greatly outweighed that of single women (haemorrhage 93.5%/6.5%, infectious diseases 89.3%/10.7%, miscellaneous 82.9%/17.1% and non-infectious diseases 82.7%/17.3%) except for abortion which had comparable differences (57.8% and 42.2%) between married and single women respectively.

Table 4 and Table 5 represent crude and adjusted odds ratio for the different groups with respect to the cause of death. Married women had a significantly higher risk of dying from haemorrhage, compared with single women (aOR = 2.7, 95% CI = 1.2-5.7). On the contrary, married women showed a significantly reduced risk of dying from abortion compared to single women (aOR = 0.2, 95%CI = 0.1-0.4). Women aged 35-39years had a significantly higher risk of dying from haemorrhage (aOR 2.6, 95%CI = 1.4-4.9), whereas they were at a lower risk of dying from abortion (aOR 0.3, 95% CI = 0.1-0.7) compared to their younger counterparts (Table 5). The results of the logistic regression analysis revealed a peculiar trend with respect to age and infectious diseases. The risk of dying from infectious diseases seems to decrease with increasing age (see Table 4 and 5). In addition, the risk of dying from miscellaneous causes increased with increasing age (Table 5).

**Table 4:** Crude Odds Ratio(OR) and 95% Confidence Interval of causes of maternal mortality according to age group, educational level, regional difference and marital status of 602 women in the reproductive age 15-49 years

Variables	Haemorrhage(*Ante partum and Postpartum*) OR (95%CI)	Other Infectious Diseases OR(95%CI)	Abortion(*Medical, Attempted, failed, other, unspecified*) OR (95%CI)	Miscellaneous OR (95%CI)	Other non-Infectious diseases OR (95%CI)
**Age group(years)**					
12-14	-	-	*	-	-
15-19	0.6(0.2-1.5)	0.6(0.3-1.5)	1.6(0.8-3.3)	1.6(0.6-4.2)	0.9(0.4-2.3)
20-24	1(Ref.)	1(Ref.)	1(Ref.)	1(Ref.)	1(Ref.)
25-29	1.3(0.7-2.4)	0.6(0.3-1.2)	0.4(0.2-0.8)	.3(0.6-3.1)	1.4(0.7-2.7)
30-34	1.7(0.9-3.3)	0.6(0.3-1.1)	0.6(0.3-1.2)	1.8(0.8-4.6)	0.5(0.2-1.2)
35-39	2.7(1.4-5.0)	0.5(0.2-1.0)	0.2(0.1-0.6)	2.0(0.9-4.6)	0.7(0.3-1.6)
40-44	1.3(0.6-3.0)	0.4(0.1-1.1)	0.6(0.2-1.5)	3.5(1.5-8.7)	0.6(0.2-1.8)
45-49	0.7(0.2-3.5)	-	2.1(0.7-6.8)	0.8(0.1-6.3)	1.0(0.2-4.6)
**Educational level**					
Never Attended	1.1(0.3-4.3)	2.6(0.3-20.6)	1.5(0.2-12.0)	0.9(0.2-4.1)	1.0(0.2-4.9)
Basic Education	0.9(0.3-3.5)	1.6(0.2-12.8)	2.0(0.3-15.7)	0.9(0.2-4.1)	0.6(0.1-3.0)
Senior High Sch.	0.8(0.2-3.4)	1.9(0.2-16.7)	2.9(0.3-24.6)	0.9(0.2-4.7)	0.7(0.1-4.0)
Tertiary/Higher	1(Ref.)	1(Ref.)	1(Ref.)	1(Ref.)	1(Ref.)
**Residence**					
Urban	1(Ref.)	1(Ref.)	1(Ref.)	1(Ref.)	1(Ref.)
Rural	1.3(0.9-2.0)	1.1(0.7-1.7)	1.1(0.7-1.7)	0.9(.6-1.5)	1.1(0.7-1.9)
**Marital Status**					
Single	1(Ref.)	1(Ref.)	1(Ref.)	1(Ref.)	1(Ref.)
Married	3.3(1.6-6.8)	1.7(0.8-3.5)	0.2(0.1-0.3)	0.9(0.5-1.7)	0.9(0.5-1.7)

*The age group 12-14years were left out because there was not enough data (only 2cases) to be included in the model for logistic regression analysis.

- No data

**Table 5:** Adjusted Odds Ratio (each variable adjusted for the rest in one model*) and 95% Confidence Interval of causes of maternal mortality according to age group, educational level, regional difference and marital status of 602 women in the reproductive age 15-49 years

Variables	Haemorrhage *(Ante partum and Postpartum*) OR (95%CI)	Other Infectious Diseases OR (95%CI)	Abortion(*Medical, Attempted, failed, other, unspecified*) OR (95%CI)	Miscellaneous OR(95%CI)	Other non-Infectious diseases OR(95%CI)
**Age group(years)**					
12-14*	-	-	μ	-	**-**
15-19	0.9(0.3-2.3)	0.9(0.4-2.2)	0.8(0.4-1.9)	1.4(0.5-3.9)	0.9(0.4-2.3)
20-24	1(Ref.)	1(Ref.)	1(Ref.)	1(Ref.)	1(Ref.)
25-29	1.3(0.7-2.4)	0.6(0.3-1.2)	0.4(0.2-0.9)	1.3(0.6-3.1)	1.4(0.7-2.7)
30-34	1.7(0.9-3.2)	0.5(0.2-1.1)	0.7(0.3-1.4)	1.8(0.8-4.2)	0.5(0.2-1.2)
35-39	2.6(1.4-4.9)	0.4(0.2-0.9)	0.3(0.1-0.7)	2.1(0.9-4.8)	0.7(0.3-1.5)
40-44	1.2(0.5-2.8)	0.3(0.1-1.0)	0.8(0.3-2.1)	3.7(1.5-9.2)	0.6(0.2-1.7)
45-49	0.7(0.1-3.5)	-	2.6(0.8-8.8)	0.8(0.1-6.3)	1.0(0.2-4.7)
**Educational level**					
Never Attended	1.2(0.3-4.6)	2.3(0.3-18.9)	0.8(0.1-7.0)	1.1(0.2-5.7)	0.9(0.2-4.6)
Basic Education	1.1(0.3-4.3)	1.4(0.2-11.1	0.9(0.1-7.8)	1.2(0.2-5.8)	0.5(0.1-2.6)
Senior High Sch.	1.0(0.2-4.3)	1.5(0.2-13.4)	1.7(0.2-15.7)	1.2(0.2-6.9)	0.6(0.1-3.3)
Tertiary/Higher	1(Ref.)	1(Ref.)	1(Ref.)	1(Ref.)	1(Ref.)
**Residence**					
Urban	1(Ref.)	1(Ref.)	1(Ref.)	1(Ref.)	1(Ref.)
Rural	1.3(0.8-2.1)	0.9(0.5-1.5)	1.3(0.7-2.2)	0.9(0.6-1.6)	1.1(0.6-1.8)
**Marital Status**					
Single	1(Ref.)	1(Ref.)	1(Ref.)	1(Ref.)	1(Ref.)
Married	2.7(1.2-5.7)	2.0(0.9-4.5)	0.2(0.1-0.4)	0.8(0.4-1.6)	0.9(0.5-1.9)

* Age *(adjusted for education, regional difference and marital status)*

* Education *(adjusted for age, regional difference and marital status)*

* Residence *(adjusted for age, education and marital status)*

* Marital status *(adjusted for age, education and regional difference)*

μ: *the age group 12-14years were left out because there was not enough data (only 2cases) to be included in the model for logistic regression analysis.*

## Discussion

### Main results

The study revealed significant discrepancies in the causes of maternal mortality that should not be underestimated. Haemorrhage was revealed as the highest cause of maternal mortality and most of those who died from haemorrhage were in the age range of 35-39 years. Previous studies also found that older mothers were at higher risk of obstetric haemorrhage compared to their younger counterparts [21,22]. This study also found that married women had higher risk of dying from haemorrhage compared to their single counterparts. Reasons for this are unknown to this study. Therefore as reasons remain to be explored; we cannot ignore the fact that when making health policies, interventions, and resource allocations to reduce maternal mortalities in Ghana; haemorrhage should be given the first priority especially in older and married women.

Conversely, married women had lower risk of dying from abortion-related causes compared to single women. This is supported by studies in other African countries that reported single women were more likely to induce abortion than married women [23,24]. In Ghana abortion is legally permitted in cases of rape, incest, risk to the pregnant woman's life, or injury to her physical or mental health and where there is substantial risk that the child may suffer from or later develop serious physical abnormalities or diseases [25]. Based on the existing law, single women who become pregnant might be seeking illegal assistance to abort the pregnancy. In this case, they expose themselves to a high-risk procedure which could explain the increased risk. In addition, pregnancy and childbirth among single women are highly stigmatized in Ghana [18]. This could be a possible explanation as to why single mothers are more likely to die from abortion related causes than married women. Therefore, a better understanding of both the formal and informal contexts of abortions for single women would give Ghana a leading edge to prevent many future maternal deaths.

Data from the study also presents a discrepancy in abortion related deaths with respect to age. The logistic regression analysis found that the risk of dying from abortion decreases as maternal age increases. The odds of dying from abortion were higher for women younger than 25 years old (Table 4 and Table 5). It has also been found in another study that more than half of all unsafe abortions happen in women of less than 25 years of age [26]. Hence, it is plausible that young women are at a higher risk of dying from abortion related causes compared to older women. A review of WHO sponsored case-studies in developing countries [26] summarised the determinants of unsafe abortion in this region; the review states that women's contraceptive choice and practice is an important personal determinant in the decision to terminate a pregnancy, which in turn leads to unsafe abortions. According to the review, attitude to contraception is negative among young adolescents and uptake of it is low [26]. Therefore the ever increasing gap between menarche and marriage in Africa, coupled with high levels of sexual activity among the youth [26] results in a higher risk of unwanted pregnancies and consequently abortion becomes an option. This may be one possible explanation as to why younger women are at a higher risk for unwanted pregnancies and consequently the higher risk of death due to unsafe abortion. One other reason may be that young women might identify pregnancies late, which increases the tendency for abortions to result in complications and death. As women grow older, they are threatened by menopause and childlessness and this gives them reason to keep pregnancies even if they are unplanned [24]. It should be noted that even though undercover abortions are high in some parts of Ghana, abortion is still thought of as an abominable practice in these areas [19]. Therefore those who try to induce abortion leave no trace of the act for the public to realise [18]. We recommend that women should be encouraged to seek early treatment for complications of induced abortion. The aforementioned review also outlines other factors that determine the safety of induced abortions in developing countries. These are services, social, economic, religious and policy factors [26]. These factors directly influence the decision making process. As a result, there is a pressing need for policy makers in Ghana to be aware of the inequalities that characterise deaths due to abortion in Ghana.

This study also found a variation in pregnancy-related deaths due to infectious diseases. The risk of dying from infectious diseases was high in younger women. Interestingly, malaria was also found in this study as the single highest cause of deaths (53.6%) due to infectious diseases among pregnant women in Ghana, distantly followed by viral hepatitis (13.1%). Malaria is known to be highly prevalent in pregnant women in Ghana and co-infections with intestinal helminths are more likely in the younger age group [27]. Given that co-infection can aggravate the complications of malaria, this provides a plausible explanation as to why younger women have an increased likelihood of dying from infectious diseases during pregnancy.

Risk of maternal deaths due to miscellaneous causes generally increased with age. These miscellaneous causes comprised mainly deaths from unspecified causes, rupture of uterus, complications of obstetric surgery, embolism, complications of anaesthesia and other complications of pregnancy, childbirth and the puerperium. This indicates that pregnancies are less safe as women grow older. A study conducted in Bangladesh [28] found similarly that pregnancies are significantly more dangerous in women beyond 35 years of age than in younger counterparts. Often information on miscellaneous causes of death, such as surgery, anaesthesia and postpartum care, documented by most district hospitals in Ghana are inadequate [29]. The inconsistency makes it difficult to investigate such causes of death among various socio-demographic subgroups in the Ghanaian population. This study therefore recommends that health workers at different levels in the healthcare system provide comprehensive information including socio-demographic characteristics on patients who die from complications of anaesthesia, obstetric surgery, embolism, rupture of the uterus and postpartum care. This will help to shed more light on variations in such causes of maternal deaths and consequently devise appropriate strategies to solve them.

### Methodological considerations

Secondary data presents with its own strengths and limitations. The major strengths in this study were the large sample size and the representative nature of the study population. This is known to increase precision of estimates of study subgroups [30]. Also, having an available dataset with a large representative sample helped researchers to focus on analysis and interpretation of the data. One common limitation usually encountered with the use of existing datasets is how best to analyze the research question within the available data [30]. In this study, all the variables that needed to be analysed in order to answer the research question were available, thus, this problem was not encountered.

Some inconsistencies in the distribution of households by cluster numbers were shown in Phase I of data collection. This could have been due to improper validation of case identifiers in the Phase I household questionnaires. Although, obvious typing errors were corrected, some could have still remained in the final data file. These are unfortunate but unlikely to bias the Phase I data on aggregate. Biases that could have risen out of recoding of variables were eliminated by cross-tabulating recoded variables with original variables for each recoded variable. This helped to ensure that no variable was wrongly recoded.

Another possible limitation could be the use of verbal autopsy questionnaires. Verbal autopsy requires skilled field based personal to collect data and office based personal to assess causes of death, code and analyse data. In addition, not all the causes listed on medical certificates can be captured with verbal autopsy [31]. The substantial expertise and resources available to the Ghana Statistical Service and its partners helped to ensure accuracy in data collection and data entry, thus minimising biases. There could have been a potential non-differential misclassification of the causes of death, however, several steps were taken to minimise this bias. First of all, causes of death were defined using sensitive and specific ICD 10 codes. Secondly, for some causes of death identified from verbal autopsy questionnaires, information was cross checked with death certificates, post mortem results, burial certificates, burial permits, antenatal homecare cards, hospital prescription forms, treatment cards, hospital discharge forms, laboratory results, community registers and other hospital documents. Despite this effort, some causes could not be verified due to lack of documentation.

## Conclusions

In conclusion, the study shows evidence of variations in the causes of maternal mortality among different socio-demographic subgroups that should not be overlooked. It shows that interventions aimed at combating the high maternal mortality ratio in Ghana should be both cause-specific as well as target specific. An example is an intervention targeted at reducing haemorrhage-related deaths in married women in Ghana or an intervention aimed at reducing abortion-related mortalities in women aged 25years and below. Further research is recommended to establish specific reasons for these variations, especially in the high-risk subgroups. The variations found out in this study could guide health authorities and policymakers when designing health interventions and policies to reduce maternal mortality in different socio-demographic subgroups.

## Competing interests

The authors declare that they have no competing interests.

## Authors' contributions

BOA was involved in study conceptualisation, data analysis, interpretation of results and drafting of the manuscript. KMM participated in designing the study, data analysis, drafting and review of the manuscript. MS also participated in data analysis, interpretation of results, drafting and critical review of the manuscript. GM contributed to the conception and design of the study and also helped to critically revise the content of the manuscript. All authors read and approved the final manuscript.

## Pre-publication history

The pre-publication history for this paper can be accessed here:

http://www.biomedcentral.com/1471-2458/11/159/prepub

## References

[B1] UN Preventable Maternal Mortality and Morbidity and Human Rights General Assembly 2009 Geneva: Human Rights Council

[B2] WHO UNICEF UNFPA The World Bank Trends in maternal mortality 1990-2008: estimates developed by WHO, UNICEF, UNFPA and The World Bank 2010 Geneva: World Health Organization

[B3] Hogan MC Foreman KJ Naghavi M Ahn SY Wang M Makela SM Lopez AD Lozano R Murray CJ Maternal mortality for 181 countries, 1980--2008: a systematic analysis of progress towards Millennium Development Goal 5 The Lancet 2010 375 9726 1609 1623 10.1016/S0140-6736(10)60518-1 20382417

[B4] Thonneau PF Matsudai T Alihonou E De Souza J Faye O Moreau JC Djanhan Y Welffens-Ekra C Goyaux N Distribution of causes of maternal mortality during delivery and post-partum: results of an African multicentre hospital-based study European Journal of Obstetrics Gynecology and Reproductive Biology 2004 114 2 150 154 10.1016/j.ejogrb.2003.12.004 15140507

[B5] Chandramohan D Rodrigues LC Maude GH Hayes RJ The validity of verbal autopsies for assessing the causes of institutional maternal death Stud Fam Plan 1998 29 4 414 422 10.2307/172253 9919634

[B6] Ordi J Ismail MR Carrilho C Romagosa C Osman N Machungo F Bombi JA Balasch J Alonso PL Menendez C Clinico-Pathological Discrepancies in the Diagnosis of Causes of Maternal Death in Sub-Saharan Africa: Retrospective Analysis PLos Med 2009 6 2 174 180 10.1371/journal.pmed.1000036 PMC2646780 19243215

[B7] A synthesis report, National Consultative meeting on the reduction of Maternal Mortality in Ghana http://www.moh-ghana.org/UploadFiles/Publications/SynthesisReport-MDG5090825082818.pdf

[B8] Zakariah AY Alexander S Roosmalen Jv Kwawukume EY Maternal mortality in the Greater Accra region in Ghana: assessing completeness of registration and data quality Acta Obstetricia et Gynecologica Scandinavica 2006 85 12 1436 1441 10.1080/00016340601040902 17260218

[B9] WHO World Health Statistics Health Status: Mortality 2006 Geneva: World Health Organization

[B10] WHO World Health Statistics Health-related Millennium Development Goals 2009 Geneva: World Health Organization

[B11] Ghana Statistical Services(GSS) Ghana Health Service(GHS) Macro International Ghana Maternal Health Survey 2007 2009 Calverton, Maryland, USA: GSS, GHS, and Macro International

[B12] Graham WJ Foster LB Davidson L Hauke E Campbell OMR Measuring progress in reducing maternal mortality Best Pract Res Clin Obstet Gynaecol 2008 22 3 425 445 10.1016/j.bpobgyn.2007.12.001 18308640

[B13] Mills S Williams JE Wak G Hodgson A Maternal Mortality Decline in the Kassena-Nankana District of Northern Ghana Matern Child Health J 2008 12 5 577 585 10.1007/s10995-007-0289-x 17957459

[B14] Geelhoed DW Visser LE Asare K van Leeuwen JHS van Roosmalen J Trends in maternal mortality: a 13-year hospital-based study in rural Ghana European Journal of Obstetrics Gynecology and Reproductive Biology 2003 107 2 135 139 10.1016/S0301-2115(02)00224-5 12648857

[B15] Zakariah AY Alexander S Roosmalen Jv Buekens P Kwawukume EY Frimpong P Reproductive age mortality survey (RAMOS) in Accra, Ghana Reproductive Health 2009 6 1 7 10.1186/1742-4755-6-7 19497092 PMC2694771

[B16] Witter S Arhinfu DK Kusi A Zakariah-Akoto S The experience of Ghana in implementing a user fee exemption policy to provide free delivery care Reproductive Health Matters 2007 15 61 71 10.1016/S0968-8080(07)30325-X 17938071

[B17] Cross S Bell JS Graham WJ What you count is what you target: the implications of maternal death classification for tracking progress towards reducing maternal mortality in developing countries Bulletin of the World Health Organization 2010 88 2 147 153 10.2471/BLT.09.063537 20428372 PMC2814479

[B18] Hill ZE Tawiah-Agyemang C Kirkwood B The Context of Informal Abortions in Rural Ghana J Womens Health 2009 18 12 2017 2022 10.1089/jwh.2008.1123 20044865

[B19] Baiden F Amponsa-Achiano K Oduro AR Mensah TA Baiden R Hodgson A Unmet need for essential obstetric services in a rural district in northern Ghana: Complications of unsafe abortions remain a major cause of mortality Public Health 2006 120 5 421 426 10.1016/j.puhe.2005.12.004 16549080

[B20] WHO ICD-10, International statistical classification of diseases and related health problems: Tenth Revision ICD-10, International statistical classification of diseases and related health problems: Tenth Revision. Volume 2 2004 Geneva: WHO

[B21] de Vienne CM Creveuil C Dreyfus M Does young maternal age increase the risk of adverse obstetric, fetal and neonatal outcomes: A cohort study European Journal of Obstetrics & Gynecology and Reproductive Biology 2009 147 2 151 156 10.1016/j.ejogrb.2009.08.006 19733429

[B22] Al-Zirqi I Vangen S Forsen L Stray-Pedersen B Prevalence and risk factors of severe obstetric haemorrhage Bjog 2008 115 10 1265 1272 18715412 10.1111/j.1471-0528.2008.01859.x

[B23] Bankole A Singh S Haas T Characteristics of women who obtain induced abortion: A worldwide review Int Fam Plan Perspect 1999 25 2 68 77 10.2307/2991944

[B24] Calves AE Abortion risk and decisionmaking among young people in urban Cameroon Stud Fam Plan 2002 33 3 249 260 10.1111/j.1728-4465.2002.00249.x 12385086

[B25] Morhee R Morhee E Overview of the law and availability of abortion services in Ghana Ghana Med J 2006 40 3 80 86 17299572 10.4314/gmj.v40i3.55256 PMC1790853

[B26] Warriner IK Shah IH (Eds.) Preventing Unsafe Abortion and its Consequences: Priorities for Research and Action 2006 New York: Guttmacher Institute

[B27] Yatich NJ Yi J Agbenyega T Turpin A Rayner JC Stiles JK Ellis WO Funkhouser E Ehiri JE Williams JH Malaria and Intestinal Helminth Co-infection Among Pregnant Women in Ghana: Prevalence and Risk Factors Am J Trop Med Hyg 2009 80 6 896 901 19478245

[B28] Khan AR Jahan FA Begum SF Maternal mortality in rural Bangladesh: the Jamalpur District Stud Fam Plann 1986 17 1 7 12 10.2307/1966950 3485842

[B29] Hussein J D'Ambruoso L Armar-Klemesu M Achadi E Arhinful D Izati Y Ansong-Tornui J Confidential inquiries into maternal deaths: Modifications and adaptations in Ghana and Indonesia Int J Gynecol Obstet 2009 106 1 80 84 10.1016/j.ijgo.2009.04.007 19428011

[B30] HOFFERTH SL Secondary Data Analysis in Family Research Journal of Marriage and the Family 2005 67 4 891 907 10.1111/j.1741-3737.2005.00182.x

[B31] Garenne M Fauveau V Potential and limits of verbal autopsies Bull World Health Organ 2006 84 3 164 10.2471/BLT.05.029124 16583068 PMC2627293

